# Patient perceptions of the side-effects of chemotherapy: the influence of 5HT3 antagonists.

**DOI:** 10.1038/bjc.1997.507

**Published:** 1997

**Authors:** M. de Boer-Dennert, R. de Wit, P. I. Schmitz, J. Djontono, V. v Beurden, G. Stoter, J. Verweij

**Affiliations:** Department of Medical Oncology, Rotterdam Cancer Institute (Daniel den Hoed Kliniek) and University Hospital, The Netherlands.

## Abstract

In 1983, Coates conducted a survey that ranked the side-effects perceived by patients receiving chemotherapy in the order of their severity. Vomiting and nausea were found to be the two most distressing side-effects. They have an impact on quality of life and compliance with treatment. The development of 5HT3 antagonists has been a major step forward in the prevention and treatment of chemotherapy-induced nausea and vomiting. Presently, these antiemetics are routinely used as concomitant therapy in emetogenic chemotherapy regimens. The purpose of this study was to evaluate the impact of 5HT3 antagonists on patient perceptions of the side-effects of chemotherapy. Coates' survey was replicated in patients who received 5HT3 antagonists for acute nausea and vomiting resulting from emetogenic chemotherapy. Patients received the survey to identify those physical and non-physical side-effects that they attributed to chemotherapy and were asked to rank the five most distressing side-effects. Of the 197 patients who consented to take part in the study, 181 were evaluable. Nausea, hair loss and vomiting were described as the three most distressing side-effects of chemotherapy. Eighty per cent of all the patients actually experienced nausea and 57% experienced vomiting. Hair loss appeared to be more distressing to women (P < 0.001) but, in other aspects, gender, age and marital status did not influence the ranking of the three most distressing side-effects. Constipation was ranked as 6th and was not identified as a distressing side-effect in 1983. Nausea and vomiting remain to be the first and third most distressing side-effects of chemotherapy, even though the incidence and severity of acute nausea and vomiting are now significantly reduced.


					
British Joumal of Cancer (1997) 76(8), 1055-1061
? 1997 Cancer Research Campaign

Patient perceptions of the side-effects of chemotherapy:
the influence of 5HT3 antagonists

M de Boer-Dennert1, R de Wit', PIM Schmitz2, J Djontono1, V v Beurden', G Stoterl and J Verweijl

Departments of 'Medical Oncology and 2Biometrics, Rotterdam Cancer Institute (Daniel den Hoed Kliniek) and University Hospital, Rotterdam, The Netherlands

Summary In 1983, Coates conducted a survey that ranked the side-effects perceived by patients receiving chemotherapy in the order of their
severity. Vomiting and nausea were found to be the two most distressing side-effects. They have an impact on quality of life and compliance
with treatment. The development of 5HT3 antagonists has been a major step forward in the prevention and treatment of chemotherapy-
induced nausea and vomiting. Presently, these antiemetics are routinely used as concomitant therapy in emetogenic chemotherapy
regimens. The purpose of this study was to evaluate the impact of 5HT3 antagonists on patient perceptions of the side-effects of
chemotherapy. Coates' survey was replicated in patients who received 5HT3 antagonists for acute nausea and vomiting resulting from
emetogenic chemotherapy. Patients received the survey to identify those physical and non-physical side-effects that they attributed to
chemotherapy and were asked to rank the five most distressing side-effects. Of the 197 patients who consented to take part in the study, 181
were evaluable. Nausea, hair loss and vomiting were described as the three most distressing side-effects of chemotherapy. Eighty per cent of
all the patients actually experienced nausea and 57% experienced vomiting. Hair loss appeared to be more distressing to women (P < 0.001)
but, in other aspects, gender, age and marital status did not influence the ranking of the three most distressing side-effects. Constipation was
ranked as 6th and was not identified as a distressing side-effect in 1983. Nausea and vomiting remain to be the first and third most distressing
side-effects of chemotherapy, even though the incidence and severity of acute nausea and vomiting are now significantly reduced.
Keywords: chemotherapy; nausea; vomiting; side-effects; serotonin antagonists

Nausea and vomiting have been reported by patients, nurses and
physicians as the most distressing side-effects of chemotherapy
(Coates et al, 1983; Love et al, 1989; Pritchard, 1989; Cooper,
1992; Martin, 1992; Youngblood et al, 1994). The introduction of
the 5HT3 antagonists Tropisetron, Ondansetron and Granisetron
have significantly reduced the incidence of these distressing side-
effects. Physicians and nurses have observed that 5HT3 antago-
nists have contributed to an improvement of quality of life and
compliance with treatment (Seynaeve et al, 1991a). However,
the impact of these new drugs on patients' perceptions of
chemotherapy-induced side-effects has not yet been investigated
(Manson et al, 1993). Unfortunately, some health care workers
extrapolate data on the improvement of antiemetic efficacy in the
phase of acute emesis to represent the total experience of emesis in
treated patients. They tend to believe that a decrease in the inci-
dence and severity of acute emesis reduces the patients' distress
accordingly. This might not be the case (Love et al, 1989; Bliss et
al, 1992; Schmoll, 1992; Jansen et al, 1993; de Wit et al, 1996) as
delayed emesis remains a serious problem. Therefore, we believed
a reassessment and re-ranking of the most distressing side-effects
of chemotherapy perceived by patients was warranted. In addition,
the most distressing side-effects of chemotherapy during each
course of treatment may be crucial for the planning of appropriate

Received 20 November 1996
Revised 14 April 1997

Accepted 18April 1997

Correspondence to: M de Boer-Dennert, Department of Medical Oncology,
Rotterdam Cancer Institute (Daniel den Hoed Kliniek) and University
Hospital, Groene Hilledijk 301, 3075 EA Rotterdam, The Netherlands

interventions and for identifying possible research questions
(Martin, 1992; Youngblood et al, 1994; de Wit et al, 1996).

We studied the patients' perceptions of physical and non-phys-
ical side-effects of current chemotherapy, using the questionnaire
previously used by Coates et al (1983) in an era before the intro-
duction of the 5HT3 antagonists. Patients ranked their side-effects
in order of distress. Influencing factors, such as the number of
treatment courses and patient characteristics, were also analysed
for their effect on the order of distress. The ranking of nausea and
vomiting was than compared with the results published by Coates
in 1983 and by Griffin in 1996.

PATIENTS AND METHODS

Eligibility criteria for the study included the following: age 2 18
years; treatment with emetogenic chemotherapy and concom-
mitant 5HT3 antagonists for the prevention and/or management
of acute nausea and vomiting from the first treatment cycle on.
Patients were treated with chemotherapy in the outpatient depart-
ment or were admitted to the hospital. Patients entered the study
during any cycle of chemotherapy.

For the prevention of acute nausea and vomiting, patients were
treated with either Ondansetron 8 mg or Tropisetron 5 mg as
single agent or in combination with dexamethason 10 mg. Both
were administered intravenously 15 min before the start of
chemotherapy. For delayed nausea and vomiting, various
antiemetics were prescribed.

Patients were informed by the participating physicians or the
study nurses about the objectives of the study. After consenting,
the nurse explained how the questionnaire was to be completed.
All patients gave verbal informed consent according to the rules of

1055

1056 M de Boer-Dennert et al

Table 1 Group A - physical symptoms

Table 2 Group B - non-physical symptoms

Feeling sick (nausea)
Being sick (vomiting)

Itching at injection site
Shaking all over

Change in the way things taste
Changes in how things smell

Not having regular bowel action (constipation)

Loss of liquid or frequent bowel action (diarrhoea)
Pins and needles in fingers and toes
Numbness in fingers or toes
Loss of weight
Weight gain

Increased hair growth on legs
Constantly tired

Giddiness on standing up
Loss of appetite
Sore mouth
Sore throat

Shortness of breath
Skin rash

Bruise easily

Difficulty sleeping

Pain passing water (painful urination)
Coloured urine
Ringing in ears
Deafness

General aches and pains

Tummy ache (abdominal pain)

Swollen tummy (abdominal fullness)
Periods stop

Periods become irregular
Changes in skin colour
Hot flushes

Heart beating fast (palpitations)
Headache/migraine
Loss of hair

Increased thirst

Passing more water than usual (increased urination)
Dry skin

Acne (pimples)

Increased appetite

Trouble with swallowing
Nose bleeds

Cannot taste things

Fingernails go brown

our institute. The survey was conducted using the questionnaire
used previously by Coates et al (1983). Side-effects were divided
in two groups: group A (Table 1) comprised 45 physical side-
effects and group B (Table 2) compised 28 non-physical side-
effects. It was possible to add side-effects experienced by the
patient that were not included in the listings. Patients were asked
to circle all side-effects that they attributed to their chemotherapy.
Subsequently, they ranked the five most distressing side-effects
that they experienced from each group in order of severity. The
two groups of five physical and non-physical side-effects were
combined and patients then ranked the five most distressing side-
effects regardless of group. We agreed to use a self-reporting ques-
tionnaire as it allows for privacy and facilitates the disclosure of
symptoms or side-effects of a sensitive nature (Youngblood et al,
1994). For analysis of the data, we allocated 5 points to the most
distressing side-effect decreasing to 1 point for the side-effect
ranked as the least distressing. Limited demographic data were
collected; age, gender, marital status, diagnosis, prior
chemotherapy regimens, the current chemotherapy regimen and
number of treatments given, the antiemetic treatment regimen

2
3
4
5
6
7
8
9
10

11

12
13
14
15
16
17
18
19
20
21
22
23
24
25
26
27
28

Loss of sexual feeling

Loss of sexual ability (not getting aroused)
Feeling low, miserable (depression)
Thought of coming for treatment

Length of time treatment takes at the clinic
Feeling bad tempered (irritability)
Having to have an injection

Having to come to clinic rather than priVate doctor
Affects my family or partner

Feeling of not coping generally with treatment

Feeling of having to have treatment that I do not think will do any good
Feeling of having to have treatment that I do not want
Crying more often
Feeling angry

Cannot concentrate

Affects my work/home duties
Affects my social activities

Infertility (cannot have children)

Trouble finding somewhere to park near the clinic
Trouble getting to the clinic

Not having the chance to ask the doctor questions
Forget things

Not seeing the same doctor each time
Cannot get clothes to fit

Not understanding what is happening
Feeling anxious or tense

Having to wait for treatment with other patients

Feeling that the treatment is damaging my body

and response to chemotherapy. Age was grouped into less than
45 years, 45-60 years and over 60 years.

Chemotherapy regimens were grouped into cisplatin based,
doxorubicin based and those comprising neither of the two drugs.
If the statistical analysis listed two items ranked as equally
distressing, both items were allocated the next lower number in
sequence. Corrections were not made for disease-related symp-
toms in the side-effects that patients reported as relating to their
chemotherapy.

The relative severity of side-effects was analysed using
descriptive statistical methods (ranking the data) and tabulated
against the patient characteristics, diagnosis, treatment and
response. When a correlation was found with the chi-square test,
the exact test for r x c was formally performed. Patient character-
istics, diagnosis, treatment and response were combined with the
severity of each side-effect separately and formally tested with
Fisher's exact test for a 2ck table; for these analyses, severity was
expressed as a dichotomy: belonging to the five most severe side-
effects (yes/no). The usual level of 5% was used as the level of
statistical significance.

RESULTS

Two hundred patients were asked to participate in the study and
197 patients completed the questionnaire. Eight patients filled out
the questionnaire incorrectly and upon analysis eight additional
questionnaires demonstrated inconsistencies and were also
excluded from the final analysis. The characteristics of the 181
evaluable patients are shown in Table 3. Fifty-six patients were
treated in the outpatient department, 125 received their treatment
as inpatients. Chemotherapy regimens and the antiemetics used to
prevent chemotherapy-induced acute and delayed emesis are listed
in Table 4. Patients had received 1-20 courses (mean, four)
of chemotherapy at the time of completing the questionnaire;

British Journal of Cancer (1997) 76(8), 1055-1061

2
3
4
5
6
7
8
9
10
11
12
13
14
15
16
17
18
19
20
21
22
23
24
25
26
27
28
29
30
31
32
33
34
35
36
37
38
39
40
41
42
43
44
45

0 Cancer Research Campaign 1997

Patients' perception of side-effects of chemotherapy 1057

Table 3 Patient characteristics

No. of evaluable patients
Male/female

Mean age (range) (years)
Marital status

Spouse

No spouse
Alone

Not alone

Tumour types

Breast cancer

Soft-tissue sarcoma
Testicular cancer

Small-cell lung cancer (SCLC)
Head and neck cancer

Non-small-cell lung cancer
Ovarian cancer
Mesothelioma
Miscellaneous

181

101/80

50 (18-78)

141
40
24
157
50
26
20
14
13
10
9
7
32

17 patients had received prior chemotherapy. The mean number of
selected physical side-effects was eight (range 0-26) and of non-
physical side-effects was five (range 0-17). Patients ranked
nausea, hair loss and vomiting as the most distressing side-effects;
the ten most distressing side-effects are listed in Table 5. Table 6
lists the analysis of the ten most distressing side-effects by gender,
age and marital status compared with the overall ranking, which is
given in the top row of the table. Items ranked in the top ten
subanalysis that were not listed in the top ten of the overall
analysis are listed on the right side of the table. The ranking of the
four most distressing side-effects was quite consistent for gender,
age and marital status.

Compared with men, women ranked hair loss significantly
higher than vomiting (P < 0.001), and they also ranked feeling
miserable (depression), anxious or tense higher than men. Men
were more concerned by the thought of coming for treatment, the
length of time treatment takes at the clinic (P = 0.006) and by
infertility.

Infertility caused more distress in the younger patients
(P < 0.001). The ranking of effects on family and partner and of
feeling anxious or tense decreased with age, while the ranking of
constipation and of having to have an injection increased. The
thought of coming for treatment affected older patients less
(P = 0.045). Crying more often was important to the patients aged
45-60 years (P = 0.048).

Table 4 Chemotherapy given and antiemetics used

Chembtherapy

Including cisplatin

Cisplatin

CisplatinAffosfamide
BEP
VIP

Other

Including doxorubicin

DoxorubicinAffosfamide
Other

Neither cisplatin nor doxorubicin

FEC
CMF
ICE

Other
Both

Antiemetics
Day 1

Ondansetron

Ondansetron/dexamethasone
Tropisetron

Tropisetron/dexamethasone
Days 2-5

Domperidon

Metoclopramide

Zofran ? methylprednisolone
Tropisetron

Ondansetron + dexamethason
Ondansetron

Dexamethasone
Various

89
40
17
15

6
11
25
15
10
65
26
21

7
11
2

11
81
32
57

63
44
10
8
6
6
4
40

BEP: bleomycin, etoposide, cisplatin; VIP: etoposide, ifosfamide, cisplatin;

CDDP: cisplatin; VP16: etoposide; DOXO: doxorubioin; FEC: 5-fluorouracil,
epirubicin, cyclophosphamide; CMF: cyclophosphamide, methotrexate,

fluorouracil; ICE: ifosfamide, carboplatin, etoposide; Other: less than four
equal regimens.

Patients living alone were obviously less concerned about the
effects on family or partner. Constipation was ranked lower in
patients living alone, the influence on loss of appetite and taste was
ranked higher.

Table 7 shows the analysis by tumour type and chemotherapy
regimen. The subgroups are smaller and a wider variability was
found. The testicular cancer patients were predominantly in the

Table 5 Ten most distressing side-effects of chemotherapy

Rank         1983                                            1995

1           Being sick (vomiting)                           Feeling sick (nausea)
2           Feeling sick (nausea)                           Loss of hair

3           Loss of hair                                    Being sick (vomiting)
4           Thought of coming for treatment                 Constantly tired

5           Length of time treatment takes at the clinic (24)  Having to have an injection
6           Having to have an injection                     Constipation (-)

7           Shortness of breath (15)                        Thought of coming for treatment
8           Constantly tired                                Affects family or partner

9           Difficulty sleeping (21)                        Feeling low, miserable (depression) (14)
10           Affects family or partner                       Feeling anxious or tense (13)

Numbers in parentheses indicate the ranking number in the opposite column.

British Journal of Cancer (1997) 76(8), 1055-1061

0 Cancer Research Campaign 1997

co~~        c>
a)~~~~~~~~~1

_   Co     -   0CU

-v    2 a,o o     co

co cD --*o- D

0 0 '-          0 0

o       3_   No tl)E  c o  o  co C -

0e,     00Ma' O no o    ?  ?
C      CY)  ?  s      ?  r-  0)

0) 0)     Co         0)

00)         LO     0) y2

(D       N-N-         -      co L

CC() L c  N   CD

C  CY)     CN      C) CM
Ch CM       C)     CM C)

coCD)     CD       CD

C) CY)    N        C')
CM NN     C')      N

a

Ot T X0Qt

o~~~c _3

a) ~ ~ ~ C s  co c

04
CM

a

rz-

v-

(0)

c

CU
0

0
z

A
V

X
CM

0
'a
co

._

E

Cb
0

:  3-

co

0

( D
C

0 co

0

*C 0

c D

Co
00

*_ 0

as

- o

CO

C V

0 -0

E

-0

Ca

.W  cv)

0.
Cf )
06)
0-

C 0
Co
0 3

0

Ocr

0
CD

c 0)

*0
0.

3~ 0

00_

00

British Journal of Cancer (1997) 76(8), 1055-1061

1058 M de Boer-Dennert et al

T-            CD

N-

LO' ?     N-    O C

0

r

0
_
0

0 2

0

i m

z._

0)

CL

00

E

0 0

0
c-

CL

I.2
co

U
0.

> E

Co

0 0

Co-
._o
X

Co
00
C._
O 0

0
-J

0 0

C Z
c.
OL

LO

Cf)
N

0.
0)
a)

c

wU

0

-

CO)

E
'a
C

0)

x

0
co

>Z

a)
cn
0
>n
.0
co

0
0
0)
C
0

r-
.0

.0
?5
to

-

0

- v

C) 0
c0

x 2LL
CD)

Un   cr)

L-d

V
0)

0 Cancer Research Campaign 1997

Patients' perception of side-effects of chemotherapy 1059

o

-5

a)
co   c

S

a)   a)

in a)

E

a)

_$.  >,  ct

-~  c E

-  (D-

a)0

oc~~~~~~~~~~~c

co C  a

0.C    0

0 0 L. 0) co  O)t CD

U)
0)C

E -

co _       _
a)<x       C

.E  0)

U) CUO   .>

co  0

a)

0) t C

a) ) sC c
U- J Cl) 0

I   coD co     c0?       CY   0
o       ?   LO     0) C'4 LO CY)  r   o

0      0
0     0

E     E

C     C

E -E

D     c0   0 co

co

a)AU-a

0    oa   Va
Ca )  >E     a E

7 O        O c  C

z t5  0  =     -
0 iE 0 0 0 0  0
CD 5 < -i <-- _  C

co  C)       0 J rL <I

CD  O  t   O~~~~~~~r- C\)

CD     LO .-         0) LO
0)     N  (C        0D T

CM     lt    N)     (_ 0(0 ?       T    N.       N.     0)       co (D

I       r-     w  I ww       0) r--                        rl Orl

't        0)    0)       L      l_ LOC     C'f)

LO

C    CD

t      O  't    t   t 't    CD  t       t   C (D    t t

CN       CV )   C        C')  C')-t C'C)   C')   C')         C')   C')C')       C')C

N c\

CN - CM C N CM  CN

N4  T  C N CN CM

CM           _- CN -- _      _     _

0

-

CO)

CD 0 0)

Lo^  co  (D  0

0j  a)0 O 2)   0'

a)s  0 0 x =0

2  E D oE      O c:

0          H

R

rI

E

CD

E
cu

a)

a N

0'

0   q
o v-

CD CD

N C')
C') 't

a-0
Dt co
LO Al

British Journal of Cancer (1997) 76(8), 1055-1061

S

-

.0

-6.

0

a)
CO

, !

uz .
0
U)-

0) LO

_    c\
_- C)

0
0)
0c

N_                      CM

N.0

T-N

h 0 @

.0

if > c

.E

I- Qo 2
o

U.

0.

E c

CL

-0 0
c._
0

~C

.5 >
0 *'

CLu.

Ca-

Y

0

C)

. .2

CE
0

0
LA.

LO

LO 0)

',CT            Cf   0

E

a)
V
0
a)
'a

C
o
E

a)

Ca)
0)
CL
a
a)
a)
m
E

E

a)
Tco

a)
._.
0
.0
._cn
n
6

CL

i-

-

v
x
cis
a)

CI
a)
0
E

0 c

(D'

CD

'a)a
C

cD E

a)
.)C

a) .c
co c
.2 a)

a) -
-~0

0a

= a)

-C .

a)'-

._0
D

_ C

- a)

l)V

C Co

' -

(D ai

0a)

a)O-

0  0

a)    c
VCO)

_! C') 3
0  -a-
eC 0)e

_E V3 Cc$

c _L

a)

IC CO

0.
aa)

*a CO)

Ca 0.

.E cn e

a)0

L0 . _
a)00,

N       _- L

a) N

0 ) )   a)-
C j

W    H   E   -
6    _- o .n

O 3 2 =n

E    , En u)
11   m

-
aY)

C
c
'a
CIO

0 Cancer Research Campaign 1997

1060 M de Boer-Dennert et al

Table 8 The incidence of nausea and vomiting vs the overall top five rating

Course of treatment      Incidence (%)           Top five rated (%)       Meana

Nausea

1 +2                        76                       59                   4.1
3                           81                       76                   4.3
4                           77                       78                   3.8
5                           79                       66                   4.1
> 6                         88                       60                   4.1
Vomiting

1 + 2                       60                       48                   4.0
3                           61                       68                   3.2
4                           46                       75                   3.8
5                           52                       55                   3.5
>6                          65                       58                   3.3

aThe mean relative severity of the points allocated from 1 (least severe) to 5 (most severe).

younger age ranges. They ranked infertility as a high distressor
(P < 0.001) and feeling miserable (depression) was ranked low.
None of the head and neck cancer patients mentioned loss of hair
and none of the small-cell lung cancer (SCLC) patient listed
constipation or feeling anxious or tense as distressing side-effects.
Patients receiving doxorubicin regimens reported hair loss as
most distressing and appeared much more concerned with the
effects on their families or partner. Patients receiving cisplatin
rated the length of time that the treatment takes in the clinic as
highly distressing compared with other patients. This could be
related to the time required for pre- and post-hydration. Statistical
analysis revealed that vomiting (P = 0.04), weight gain (P = 0.01)
and hot flushes (P = 0.03) were important to patients receiving
neither cisplatin nor doxorubicin; a group predominantly
consisting of breast cancer patients as seen in Table 4. Table 7
shows the results according to tumour type, tumour response and
the number of courses. Responding patients had considerably less
anxiety and tension, and less difficulty with the thought of
coming for treatments and having a needle. They were very
concerned, however, about the effects on their families or partner.
The thought that the treatment had damaged the body was experi-
enced as highly distressing by patients who had achieved a
complete response (P < 0.001).

Analysis of the number of treatment courses given demon-
strated that the thought of coming for treatment and depression
became more distressing after multiple treatment courses, whereas
feeling anxious or tense decreased. During multiple treatment
courses, the patients' distress related to the affects on partner and
family firstly increased and then subsequently decreased. Sleeping
problems are mentioned more frequently during course 1 and 2.
Fatigue is predominant by course 3.

Table 8 shows the percentage of patients who identified nausea
and vomiting as a side-effect of their chemotherapy per course
number and, subsequently, the percentage of these patients who
included these side-effects in their overall top five ranking; in
addition, the mean severity ascribed to these side-effects is
presented.

DISCUSSION

The introduction of 5HT3 antagonists for the prevention of
chemotherapy-induced nausea and vomiting is frequently consid-
ered to be one of the most important achievements in supportive

care in the last decade. Nevertheless, physicians and other health
care workers may overestimate this achievement. The ten most
distressing side-effects of the overall groups analysis in the present
study and the results reported by Coates et al (1983) are listed in
Table 5. The objective of this study was to investigate the current
status of patient perceptions of side-effects rather than to compare
our results with those reported by Coates et al (1983), and clearly
several factors hamper such a comparison. Firstly, our patients
received more intensive chemotherapy, partly related to the avail-
ability of 5HT3 antagonists; secondly, Coates' patient population
consisted predominantly of women. Still, our results do show that,
despite the use of 5HT3 antagonists as denominator for participa-
tion to this study, patients ranked nausea and vomiting as the first
and third most distressing side-effects of chemotherapy, which is
almost similar to the results of over a decade ago (Coates et al,
1983). This is also consistent with the findings of Griffin et al
(1996) who identified nausea as the major problem in a similar
survey. Obviously, the question is why the increased ability to
prevent nausea and vomiting is not reflected in the patients'
perception of this side-effect. First of all, as has already been
suggested by others (Cooper, 1992), the frequency and/or severity
of symptoms may not be correlated with the levels of distress
expressed by the patient. For a patient, for instance, 10 days of
minor nausea may be more distressing than 1 day of severe
vomiting. Secondly, the 5HT3 antagonists have improved the
prevention and treatment of acute nausea and vomiting rather than
the delayed nausea and vomiting. Thirdly, the studies that have
been performed on the efficacy of 5HT3 antagonists over multiple
courses appear to show that their effect is not maintained (de Wit
et al, 1996). With increasing numbers of treatment cycles, there is
a progressive loss of efficacy. This may influence the patients'
perception for the whole treatment period. Table 8 demonstrates
that a high percentage of patients already experience some nausea
and vomiting during the first two courses. Nevertheless, in the first
two courses of treatment, patients ranked nausea and vomiting as
highly distressing in our study, again suggesting the relative
importance of the level of distress vs the severity and frequency of
the side-effects. Our study was not designed in a way to enable
such an analysis, but the option should be considered in further
research projects. In addition Table 8 outlines that the majority of
the patients who experience nausea and vomiting describe the
side-effects as one of the five most distressing side-effects and
rank it very highly.

British Journal of Cancer (1997) 76(8), 1055-1061

0 Cancer Research Campaign 1997

Patients' perception of side-effects of chemotherapy 1061

Hair loss remains an important distressing side-effect for
patients, which is not surprising given the fact that there are no
effective methods to circumvent this side-effect. This was also
found in the study of Griffin et al (1996). Compared with men,
women ranked hair loss as being more distressing than vomiting,
even though it is well known that they are more susceptible to the
latter. This stresses the sociopsychological relevance of hair loss to
women. Some of the gender differences with reference to hair loss
may also be explained by the fact that men are often faced with
some age-related hair loss. Patients treated with cisplatin-based
regimens also ranked hair loss as highly distressing. Cisplatin
monotherapy does not cause hair loss but, in patients participating
in this study, it was combined with drugs that are well known to
induce hair loss. Head and neck cancer patients did not report hair
loss as a distressing side-effect, as none of these patients received
any drug that causes hair loss. In comparison to the study
performed by Coates et al (1983), the length of time treatment
takes in the clinic is no longer perceived as one of the ten most
distressing factors. This may reflect the fact that in the last decade
there has been a shift from hospital admissions to outpatient treat-
ment. However, as in the Coates' study (Coates et al., 1983), the
length of time that treatment takes, as well as having to wait for
treatment, did cause more distress in men.

Constipation is a new problem compared with 1983, now being
perceived as one of the ten most distressing factors. This is a well
known side-effect of 5HT3 antagonists (Seynaeve et al, 1991b),
and the fact that all patients entered in this study received these
drugs presumably explains the high ranking of this symptom. This
may be a very important observation. Although we are able to
control nausea and vomiting better, this is not reflected in the
patients' perception of this side-effect and, moreover, this control
is achieved at the cost of other side-effects such as constipation,
which is apparently adding to the level of distress for the patient -
another reason for a careful reappraisal of the implementation and
dosing of the 5HT3 antagonists. Surprisingly, this was not identi-
fied as a distressing symptom by Griffin et al (1996).

A few distressing side-effects are no longer ranked high in the
present study compared with the previous study of Coates et al
(1983). One such side-effect is shortness of breath; the reason for
this being no longer ranked high is unclear. It may be that the
selection of patients has changed and, presently, mainly patients
with a better performance score and/or an earlier stage of disease
are treated with chemotherapy. Difficulty in sleeping is also ranked
lower. In view of the fact that feeling depressed, anxious or tense
are still ranked high, any improvement in this respect can be
excluded in trying to explain this finding. Whether night medica-
tion is either more effective or used more appropriately remains to
be elucidated.

A few other issues also warrant attention. Infertility as a result
of chemotherapy appeared to be of major concern to patients with
testicular cancer, even though it is well known that many of these
patients appear to be infertile before the start of chemotherapy
(Drasga et al, 1983). Clearly, the information given to these

patients is not sufficient in this respect. Young patients are more
anxious and are more concerned with the effects on their families
than older patients. This perhaps reflects an increased difficulty in
coping with the fact of death due to disease, for the first symptom,
and the higher probability for these patients to have important
parental tasks, for the latter.

The results of our study indicate that we should remain alert to
the patients' perception of the side-effects of chemotherapy, which
may differ from the perception of health care workers. We do not
want to underestimate the importance of the introduction of new
effective means of supportive care, such as the 5HT3 antagonists,
but would like to caution for overoptimistic interpretation of their
relevance. Only full control over nausea and vomiting will result in
a change of patients' perception. For this reason, 'complete
response' should be the main end point for studies on antiemetics
as well as for the development of new antiemetics. This is clearly
not in agreement with the optimistic results reported in the litera-
ture, in which the end point is usually major instead of complete
control.

REFERENCES

Bliss J, Robertson B and Selby P (1992) The impact of nausea and vomiting upon

quality of life measures. Br J Cancer 66(suppl. 19): S14-S23

Coates A, Abraham S, Kaye SB, Schwerbutts T, Frewin CH, Fox RH and Tattersall

MHN (1983) On the receiving end - patient perception of the side effects of
cancer chemotherapy. Eur J Cancer Clin Oncol 19: 203-208

Cooper S and Georgiou V (1992) The impact of cytotoxic chemotherapy

perspectives from patients, specialists and nurses. Eur J Cancer 28A(suppl. 1):
S36-S38

Drasga RE, Einhom LH, Wiliams SD, Patel DN and Stevens EE (1983) Fertility

after chemotherapy for testicular cancer. J Clin Oncol 1: 179-183

Griffin A, Butow P, Coates A, Childs A, Ellis P, Dunn S and Tattersall M (1996) On

the receiving end V: Patient perceptions of side effects of cancer chemotherapy
in 1996. Ann Oncol 7: 189-195

Jansen C, Halliburton P, Dibble S and Dodd HJ (1993) Family problems during

cancer chemotherapy. Oncology Nursing Forum 20: 689-696

Love R, Leventhal H, Easterling D and Nerenz DR (1989) Side effects and

emotional distress during cancer chemotherapy. Cancer 63: 604-612

Manson H, Manderino M and Johnson M (1993) Chemotherapy: thoughts and

images of patients with cancer. Oncology Nursing Forum 20: 527-532

Martin M (1992) Myths and realities of antiemetic treatment. Br J Cancer 66(suppl.

19): S46-S51

Pritchard AP and Speechley V (1989) What do nurses know about emesis? Int

Cancer Nursing News 1: 1

Schmoll H (1992) Quality of life measurements in anti-emetic trials: a discussion of

Professor Selby's papa. Br J Cancer 66(suppl. 19): S24-S25

Seynaeve C, De Mulder PHM and Verweij J (1991a) Controlling cancer

chemotherapy-induced emesis, an update. Pharm Weekbl (Sci): 13: 1-6

Seynaeve C, Verweij J and De Mulder PHM (1991b) 5HT3 receptor antagonists, a

new approach in emesis. A review of Ondansetron, Granisetron and
Tropisetron. Anti-cancer Drugs 19: 203-208

De Wit R, Schmitz P, Verweij J, De Boer-Dennert M, De Mulder PHM, Planting

ASTH, van der Burg Mel and Stoter G (1996) Analysis of cumulative

probabilities shows that the efficacy of 5HT3 antagonist prophylaxis is not
maintained. J Clin Oncol 14: 644-651

Youngblood M, Williams PD, Eyles H, Waring J and Runyon S (1994) A

comparison of two methods of assessing cancer therapy-related symptoms.
Cancer Nursing 17: 37-44

0 Cancer Research Campaign 1997                                           British Journal of Cancer (1997) 76(8), 1055-1061

				


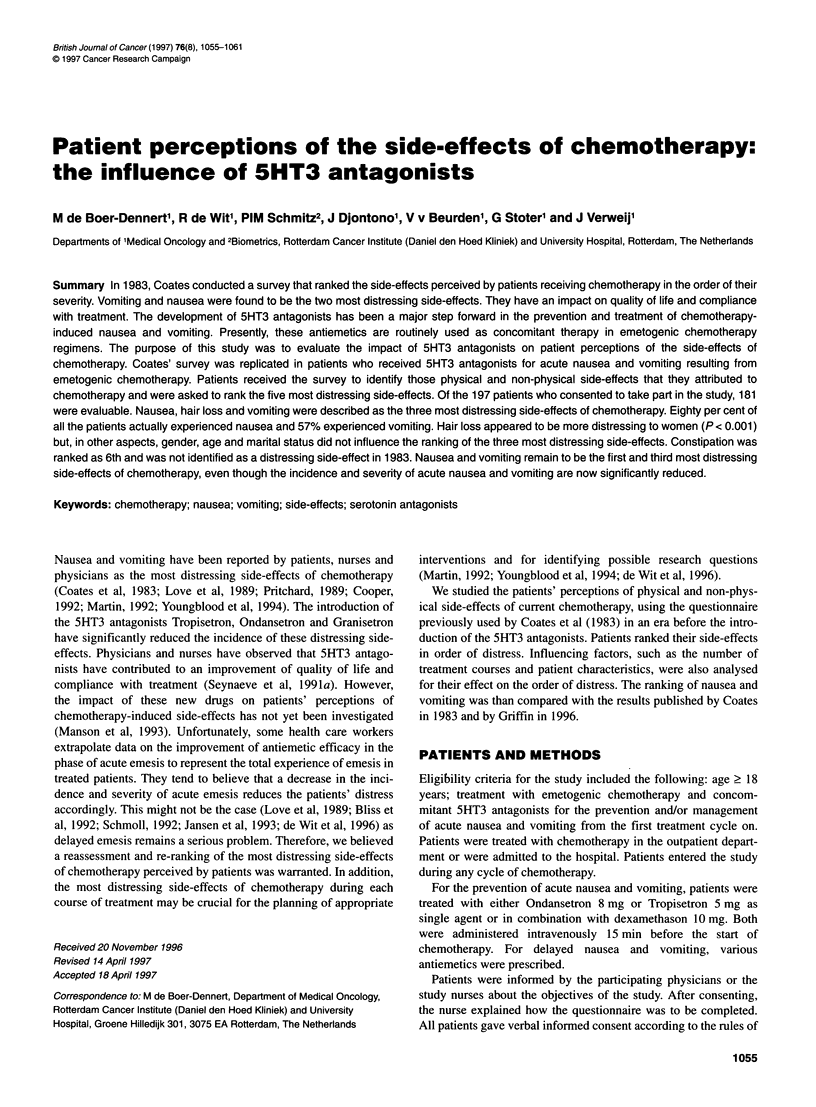

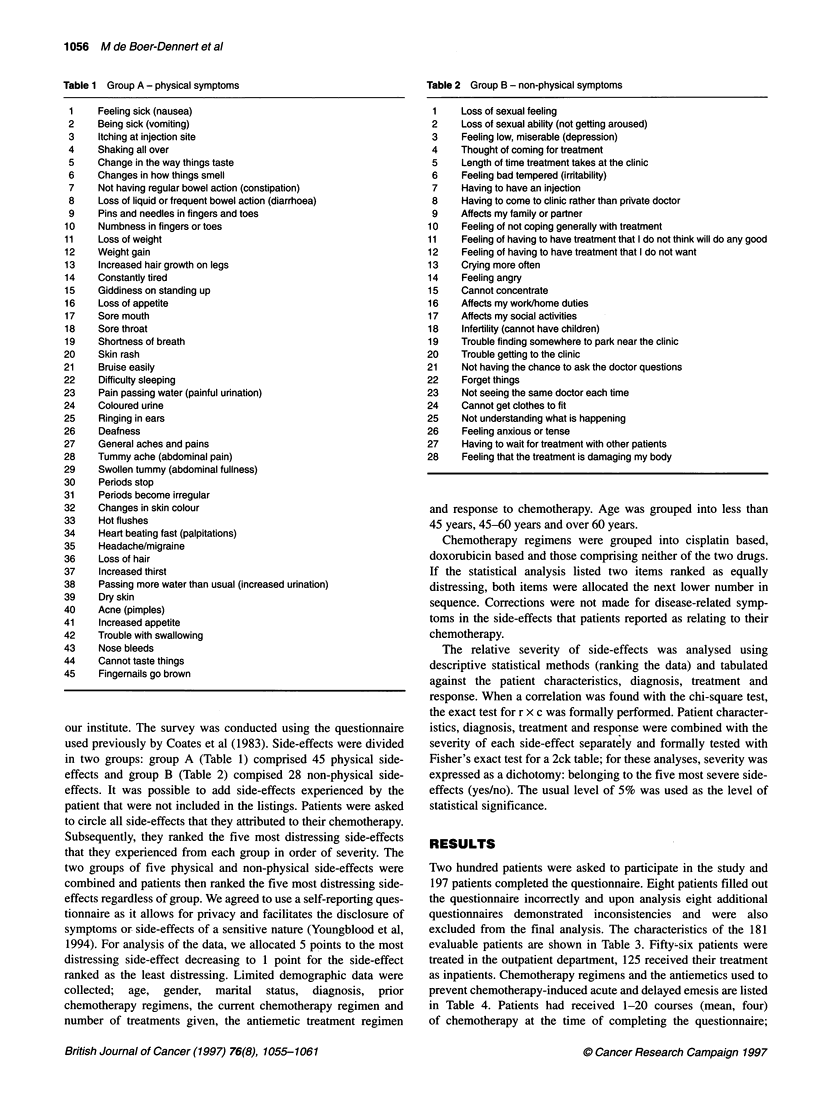

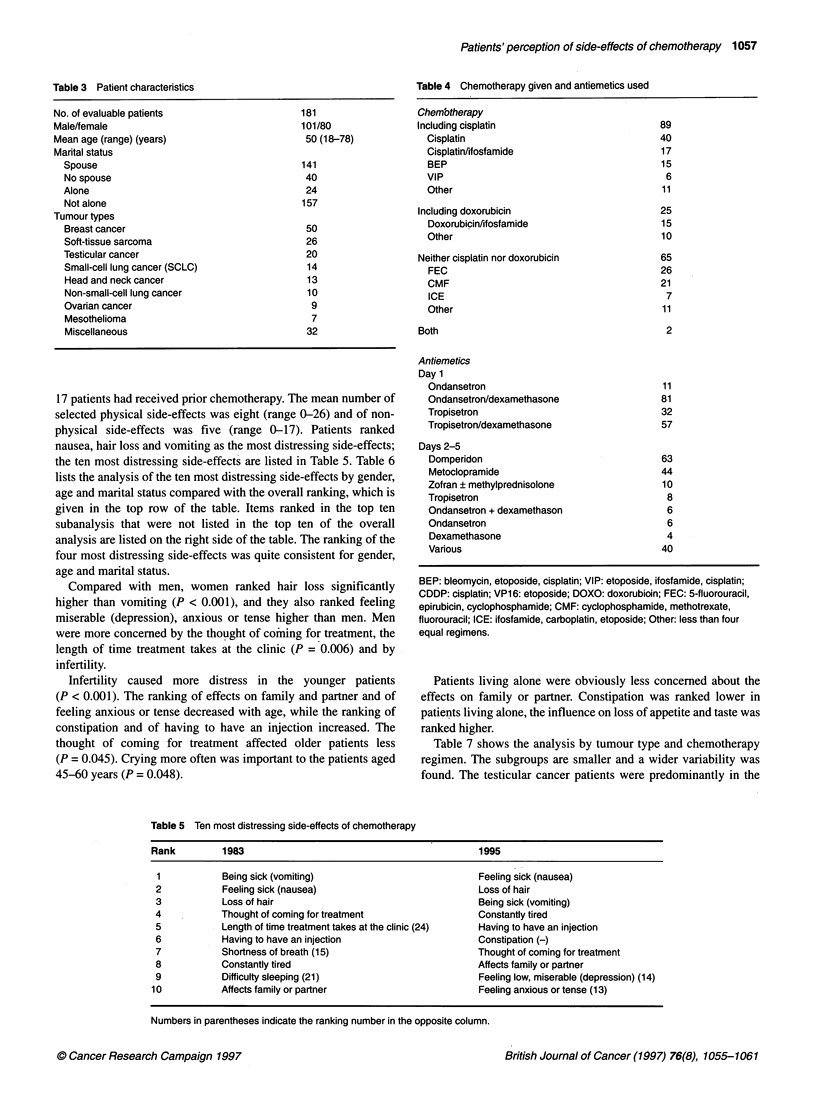

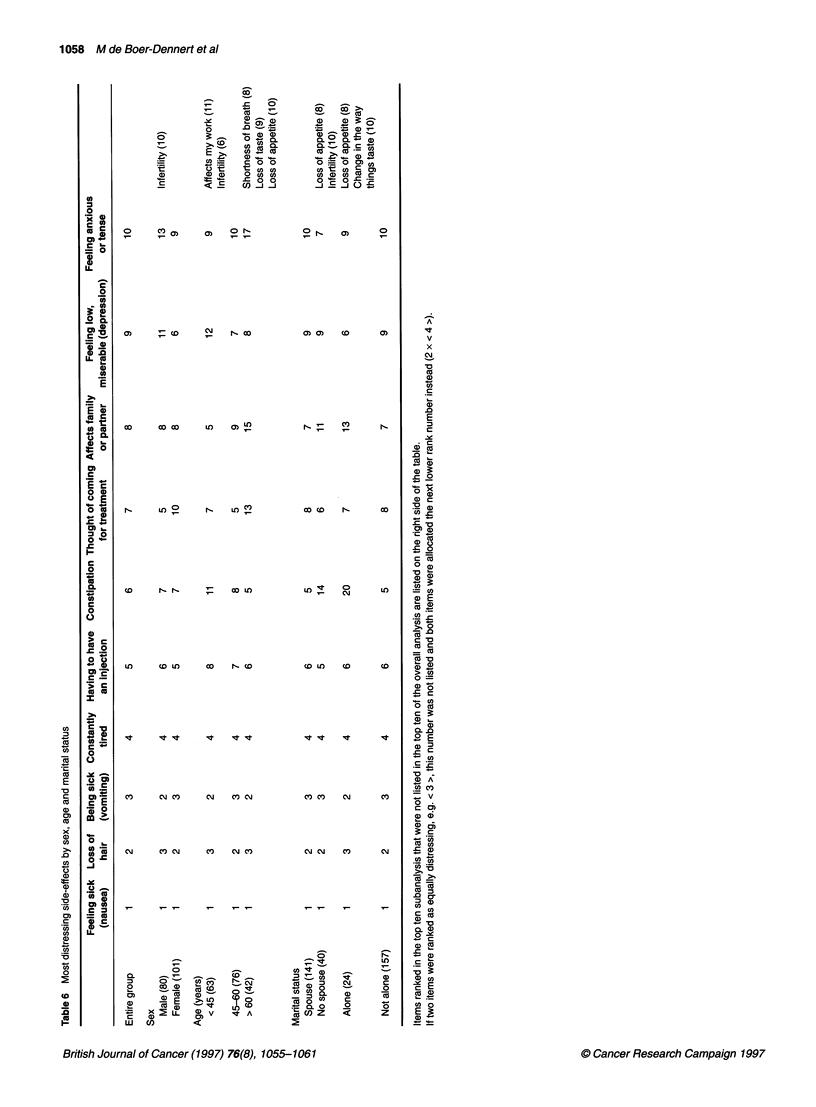

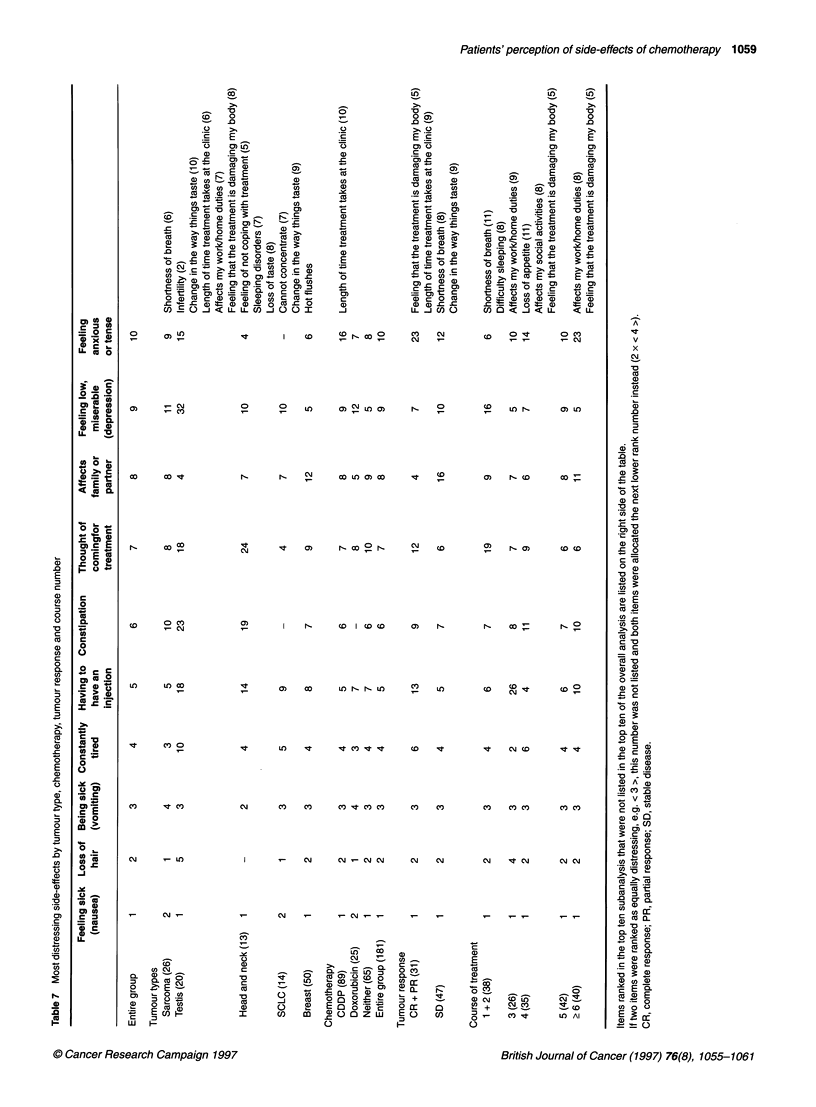

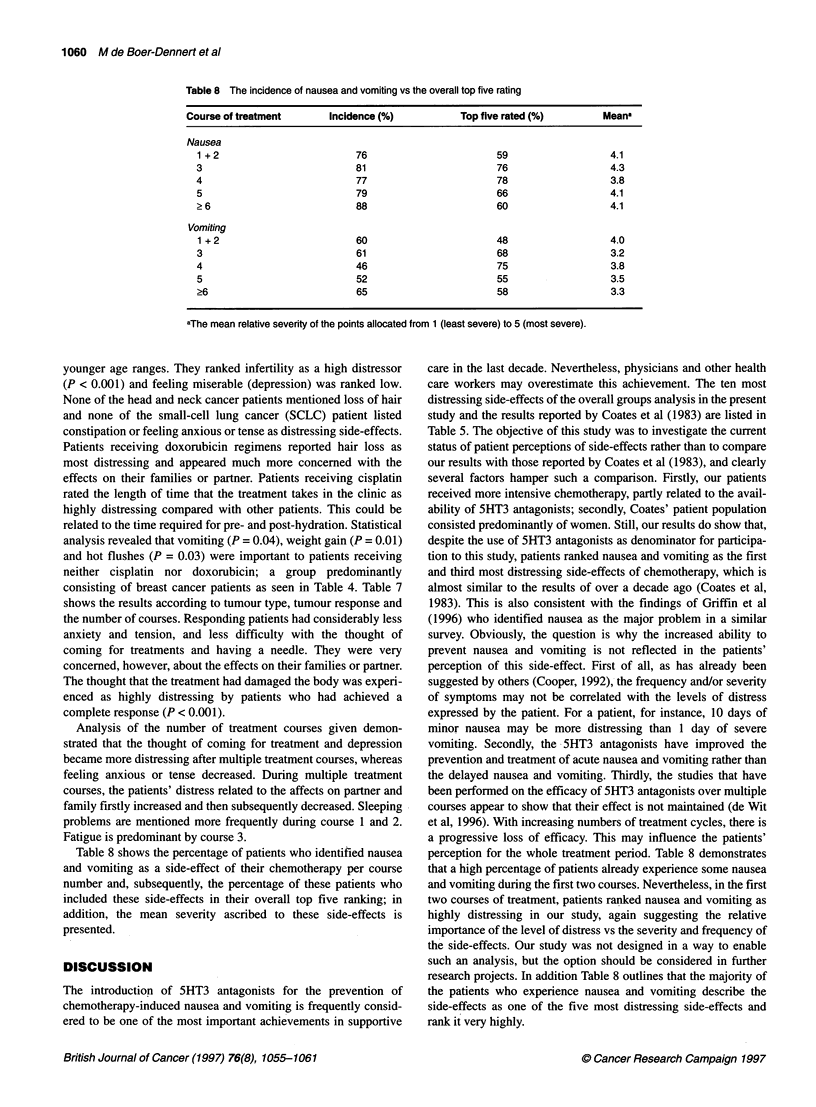

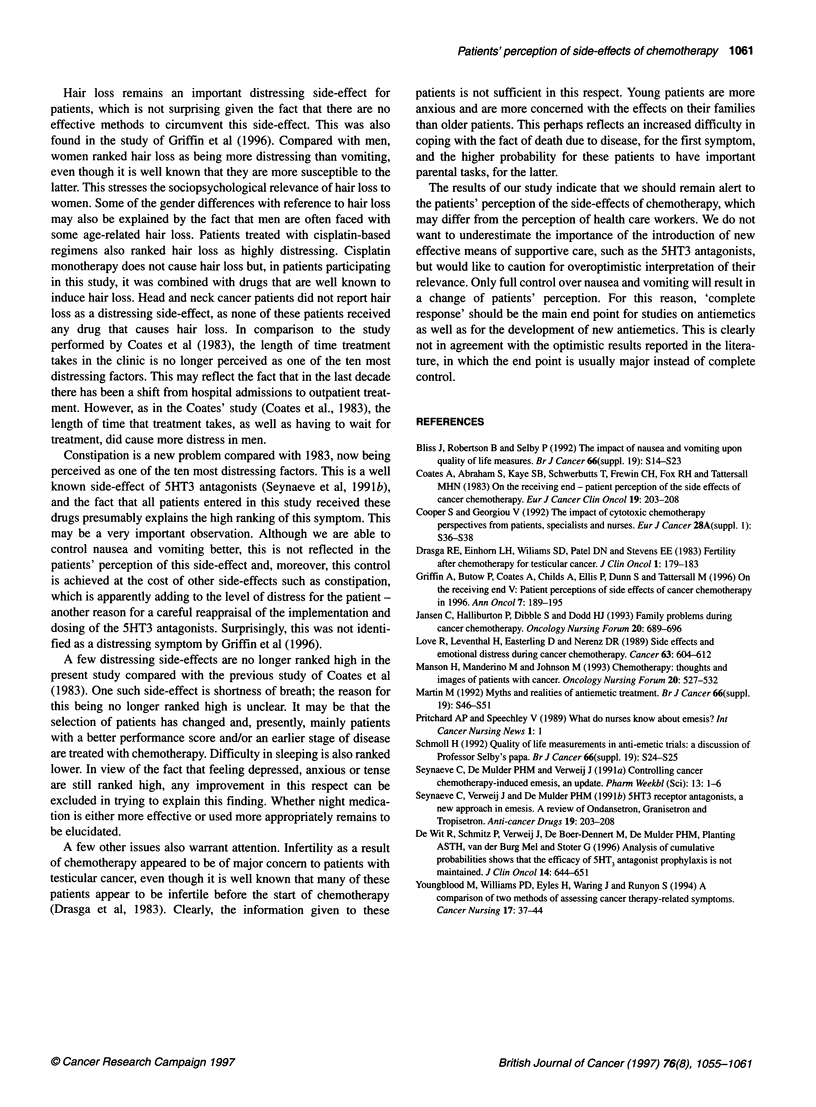

